# Imprints of Land Use History on the Cutaneous Microbiota of Mexican Cloud Forest Salamanders

**DOI:** 10.1007/s00248-025-02671-5

**Published:** 2025-12-05

**Authors:** Ángel F. Soto-Pozos, Eria A. Rebollar, Sean M. Rovito, Gabriela Parra-Olea

**Affiliations:** 1 Departamento de Zoología, Instituto de Biología, Universidad Nacional Autónoma de México, Tercer Circuito Exterior s/n, Ciudad Universitaria, México, Distrito Federal AP 70-153 México; 2 Unidad de Genómica Avanzada, Centro de Investigación y de Estudios Avanzados del Instituto Politécnico Nacional, Irapuato, Guanajuato México; 3 Unidad de Genómica Avanzada, Centro de Investigación y de Estudios Avanzados del Instituto Politécnico Nacional, Irapuato, Guanajuato México; 4 Posgrado en Ciencias Biológicas, Unidad de Posgrado, Edificio D, 1° Piso, Circuito de Posgrados, Ciudad Universitaria, Coyoacán, CDMX, C.P. 04510 México

**Keywords:** Bacterial communities, Amphibian skin bacteria, Land-use history, Secondary cloud forest, Plethodontidae

## Abstract

The cloud forest harbors the highest amphibian diversity in Mexico, particularly among plethodontid salamanders. However, the expansion of agricultural and cattle ranching activities has significantly impacted this ecosystem and their native species. Beyond direct effects on cloud forest-dwelling species, effects of land-use change on free-living and salamander skin associated bacterial assemblages are underexplored in the cloud forest and in plethodontid salamanders specifically. This study examines how historical land-use changes may influence environmental and salamander skin bacterial communities, focusing on two types of previous land-use and six sympatric plethodontid salamanders from the cloud forest. Furthermore, we explored the presence of the pathogenic fungus *Batrachochytrium dendrobatidis* (*Bd*), due to its potential interaction with salamander skin bacterial communities. We found that skin bacterial communities varied with land-use history: in habitats formerly used for agriculture salamanders exhibited higher bacterial diversity, and communities’ dispersion varied depending on the previous land-use. We found a very low *Bd* prevalence throughout the study area. Our findings suggest that bacterial communities associated with the skin of plethodontid salamanders may be influenced by land-use history in cloud forest fragments.

**Supplementary Information:**

The online version contains supplementary material available at 10.1007/s00248-025-02671-5.

## Introduction

The amphibian skin is a multifunctional organ that supports diverse microbial communities that can be essential to host survival [1, 2]. These skin-associated communities, primarily bacterial, play critical roles in various biological functions, including protection against pathogens [2, 3]. By producing specific bioactive compounds or stimulating their secretion in amphibian skin, these bacteria contribute to amphibian health and overall functionality [2]. It has been demonstrated that the structure of skin bacterial communities (SBC) is related to its functions in this organ, such as counteracting the effect of fungal pathogens like *Batrachochytrium dendrobatidis* (*Bd*) [4].

Amphibians recruit bacterial taxa to their skin from environmental bacterial communities (EBC) [5, 6]. Thus, environmental factors such as climate, seasonality, and habitat type shape the set of bacterial taxa available for skin colonization [7, 8]. On the other hand, habitat modification can disrupt both SBC and EBC by increasing physiological stress in the amphibians’ hosts, and/or altering microbial composition and diversity in environmental reservoirs [1, 9–12]. These disruptions may render amphibians more vulnerable to pathogens such as *Bd*, the causative agent of chytridiomycosis, a disease responsible for dramatic amphibian population declines worldwide [13, 14].

The Neotropical region is known for its high diversity of plethodontid salamanders [15–17]. These salamanders account for over 40% of global salamander diversity [17, 18], with more than 50% of species in this group classified as threatened by the IUCN [18]. This high level of threat is partially because many plethodontid salamanders are highly specialized, and rely on cloud forests, a highly biodiverse yet critically endangered ecosystems that is heavily impacted by deforestation and land-use change [19, 20]. Currently in Mexico, much of the cloud forest habitat consists of secondary forest remnants [21]. These secondary cloud forest remnants result primarily from regeneration following the abandonment or destination for conservation of areas used formerly for agricultural and livestock activities [19–21]. Historical differential land-use may involve changes in the composition and structure of the current bacterial communities associated with amphibians [11]. These SBC alterations are of relevance due to their potential correlation with the salamander declines caused by chytridiomycosis in these habitats in the past [14, 16, 22].

This study aims to investigate how two types of past land-use may influence the composition, structure, and diversity of EBC and SBC in tropical plethodontid salamanders inhabiting secondary forests. We studied secondary cloud forest fragments that were previously transformed into agricultural and livestock lands and we: (1) analyzed the relationship between the SBC of salamanders with their surrounding EBC in the two types of fragments; (2) examined differences in SBC of six host species in fragments previously used for livestock and agriculture; (3) analyzed the impact of two types of land-use in the past on the SBC of the widespread species *Aquiloeurycea cafetalera*; and (4) assessed the *Bd* presence in the two types of fragments. By exploring the effects of habitat alteration on amphibians’ bacterial communities, this study seeks to provide insights into amphibian conservation in recovering ecosystems.

## Methods

### Study Area

**Fig. 1:**
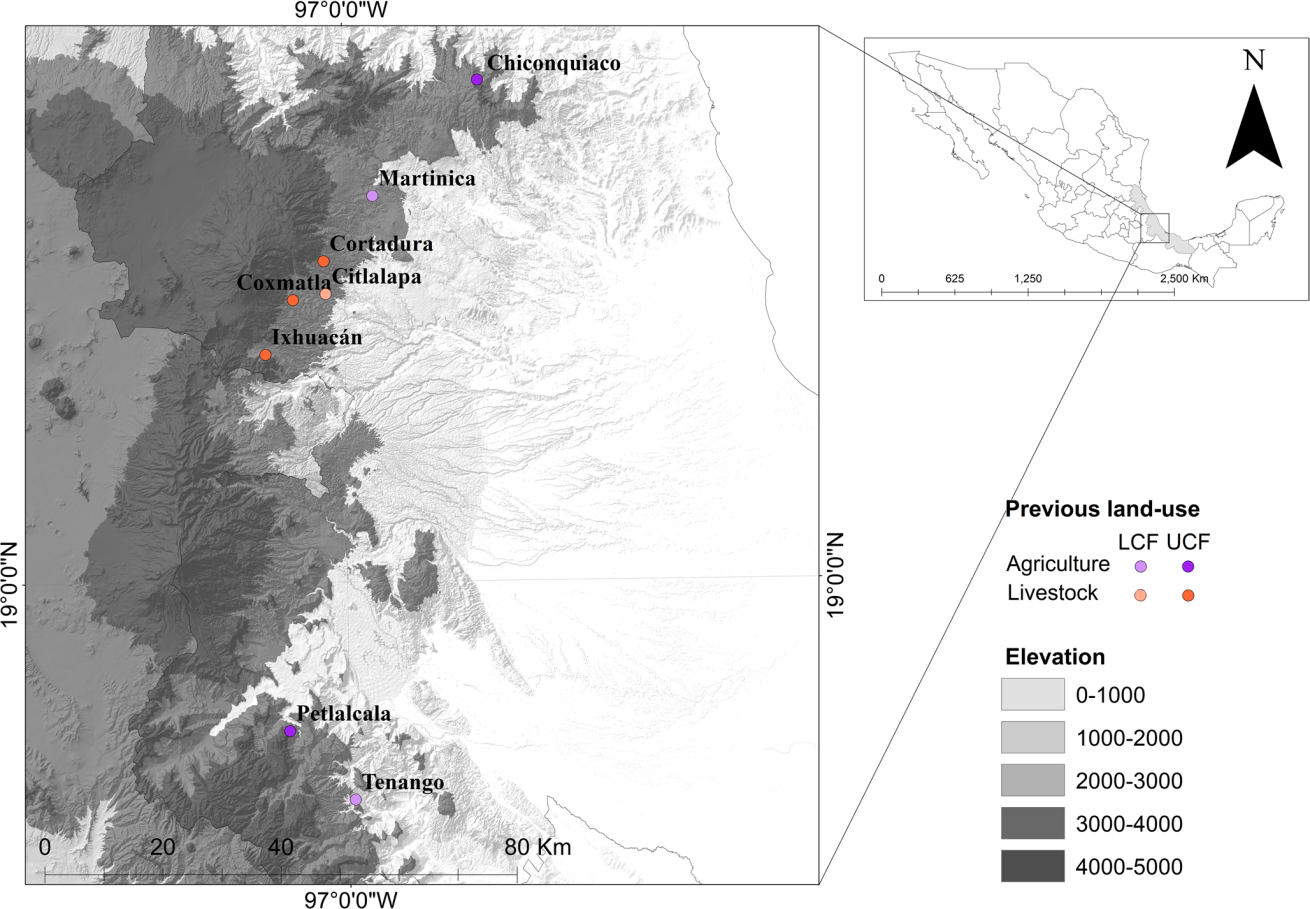
Sampling sites in the central region of Veracruz State, Mexico. Each site consists of a secondary forest fragment previously used for agriculture or livestock grazing. UCF means Upper Cloud Forest and LCF means Lower Cloud Forest

This study was conducted in secondary cloud forest fragments in central Veracruz, Mexico, between 18.65° and 19.78° N and 96.77° and 97.2° W, at elevations ranging from 1,475 to 2,130 m above sea level (m asl). In this region, the cloud forest is highly fragmented due to the advance of agricultural and ranching frontiers and is formed by remnants with variable environmental conditions [20, 21]. We selected eight sampling sites, each currently covered by secondary forest in regeneration after prior land-use for either agriculture or livestock activities (Fig. 1).

### Habitat Classification and Environmental Variables

 We classified the study sites based on historical land-use practices, using historical vegetation and land-use data from the Instituto Nacional de Estadística y Geografia [23, 24]. Secondary cloud forest fragments were grouped into two types: previously used for cattle grazing (livestock) and previously used for rainfed agriculture (agriculture). Each category included four sites, and further stratification was made, having lower (< 1,900 m a.s.l.) and upper (>1,900 m a.s.l.) cloud forest fragments (Table 1). Forest fragments have been recovering for at least 20 years. Additionally, we quantified local environmental conditions (details in Online Resource 1), to explore their relationship with the historical land-use practices.

**Table 1:** Habitat classification according to the previous land-use and elevation, and sample size (*n*) and species sampled (sp) by site after quality filtering. Upper cloud forest = sites at > 1900 m asl., lower cloud forest = sites at < 1900 m asl

Elevational category	Previous land-use	Sites	Environment *n*	Salamanders *n*/sp
Upper cloud forest	Agriculture	Chiconquiaco	7	5/1
		Petlalcala	12	14/2
	Livestock	Cortadura	15	29/3
		Coxmatla	11	20/3
		Ixhuacan	11	13/2
Lower cloud forest	Agriculture	Martinica	10	15/2
		Tenango	4	5/1
	Livestock	Citlalapa	6	8/2
Total			76	109/6

### Sampling Methods

We focused on six species belonging to four salamander genera heterogeneously distributed in the sampling sites, *Aquiloeurycea cafetalera*, *Chiropterotriton nubilus*, *Parvimolge townsendi*, *Pseudoeurycea lynchi*, *P. granitum* and *P. nigromaculata*. Among the study species, *Aquiloeurycea cafetalera* was the most widely distributed, allowing us to test the effect of habitat modification in this species separately, avoiding the bias of multiple host species.

We sampled salamanders during the rainy season in October 2019 on two consecutive nights at each site using active-search methods. Active individuals were found on terrestrial or arboreal surfaces, while inactive ones were located under moss, barks, logs, or leaf litter and within bromeliads. We identified the salamander species based on external morphology according to specialized literature.

We captured each salamander using a new plastic bag to prevent contamination and kept it for a maximum of ~ 2 h until sampling was finished. Individuals were rinsed with ~ 25 ml of sterile distilled water to eliminate transient bacteria and swabbed with a sterile rayon-tipped swab (MW113, Medical Wire & Equipment). The swab protocol included 10 strokes along the ventral surface from the neck to the vent, 10 strokes from the vent to the tail tip, and five strokes on each limb (modified from Hyatt et al., 2007). We wore new sterile nitrile gloves for each salamander to avoid cross-contamination. All salamanders were swabbed in situ to avoid the stress of translocation and released at the capture site after processing. Sample collection was performed under a permit approved by the Subsecretaria de Gestion para la Proteccion Ambiental number: SGPA/DGVS/03368/19.

To address the relationship between skin and environmental bacterial communities (SBC and EBC, respectively), we collected environmental swabs from substrates where salamanders were found, with five replicates per type of substrate. Environmental samples were taken swabbing the surfaces 40 times. Furthermore, we collected control samples by sampling site by soaking a sterile swab in the distilled water used for rinsing salamanders. Skin, environmental and control swabs were stored in 1.5 ml sterile tubes, flash-frozen in liquid nitrogen, and kept at −80 °C until further analysis.

### Amplicon Library Preparation and Sequencing 

We extracted DNA from all swab samples using the DNeasy Blood and Tissue Kit (Qiagen, Valencia, USA) following the manufacturer’s protocol, with an additional pretreatment of lysozyme incubation at 37 °C for one hour. We conducted library construction by amplifying the V4 region of the 16S rRNA gene using unique barcoded primers F515-806R and PCR conditions adapted from Caporaso et al. (2011) (Online Resource 1). PCRs were run in triplicate using distilled water as a negative control per reaction. Control swabs did not result in any amplification during library construction and thus, were not included in further sequencing. Sequencing was performed using 250 base single-end reads on an Illumina platform at the Dana-Farber Cancer Institute of Harvard University (Boston, MA, USA).

### Bioinformatic Processing

Resulting sequences were processed using Quantitative Insights Into Microbial Ecology (QIIME) version 2–2020.2.2 [25]. To maximize read quality, forward reads were trimmed to 246 bp, according to the minimum sequence length in the dataset, and filtered using a Phred quality score of 20. Amplicon sequence variants (ASVs) were identified with the DADA2 plugin, and a phylogenetic tree was generated using the q2-phylogeny plugin with FastTree [26]. Alpha and beta diversity metrics were calculated using 10,000 reads per sample according to observed ASV rarefaction curves with the q2-diversity plugin. Taxonomy was assigned using Greengenes version 13_8 [27]. We excluded ASVs that were not identified at the kingdom level or those assigned to chloroplast and mitochondria. Additionally, previous to statistical analyses, we performed a decontamination step based in concentration, which assumed that contaminants concentration is correlated with the DNA samples concentration, using decontam package [28], in R v 4.0.0 [29].

### Detection of *Batrachochytrium dendrobatidis *

 For *Batrachochytrium dendrobatidis* detection, we used a TaqMan real-time PCR assay with synthetic standards. The DNA from each skin swab sample was tested in duplicate. Synthetic standards with 10, 10^2^, 10^3^, 10^4^, and 10^5^ copies of the internal transcribed spacer (ITS1) region of haplotype Bd_Hap001 were included by plate. PCR reactions were run in 25 µL volumes using an Applied Biosystems StepOne Real-Time System following Boyle et al. (2004) conditions as follows: an initial denaturation at 95 °C for 10 min, 50 cycles at 95 °C for 15 s and 60 °C for 1 min. The raw genomic outputs from real-time PCRs were multiplied by 80 to account for dilution during extraction [30]. Samples were considered *Bd* positive if both replicates exhibited exponential amplification curve before cycle 46, and samples with undetermined results were discarded and replicated. On each plate, one negative and one positive control with known zoospores number (100 zoospores of the MexMkt *Bd* strain) were included. Zoospore genomic equivalents (GE) were calculated considering the raw genomic output in the positive control and the raw genomic average in positive samples.

### Data Analysis

We used principal component analysis (PCA) to visualize environmental variation of each type of fragments (previously used for agriculture and livestock), considering eight environmental variables: elevation, canopy cover, tree and logs density, leaf litter depth, environmental temperature and humidity, and microhabitat temperature. To analyze the influence of historical land-use in the relationship between SBC and EBC; we identified ASVs that were significantly enriched on EBC or SBC by type of fragment, using linear discriminant analysis effect size (LEfSe). We used ASV relative abundance as the predictor variable, and bacterial communities’ provenance (EBC or SBC) as the response variable, considering informative ASVs with LDA scores >2.0 [31]. Additionally, we used the approach of the analysis of compositions of microbiomes with bias correction (ANCOMBC) to identify bacterial families with differential abundance between EBC and SBC in each fragment type using the package ancombc [32] in R v 4.0.0 [29].

We calculated three alpha diversity metrics including species richness (observed ASVs), species diversity (Shannon index), and phylogenetic diversity (Faith’s index) and tested for normality using Shapiro-Wilk test (Online Resource 2). Then, we compared these metrics between SBC and EBC on each type of fragment, using t-student or Wilcoxon test, depending on whether data was normally distributed or not. Beta diversity dispersion was calculated from the weighted UniFrac distance matrix and compared between EBC and SBC by fragment type using t-student and Wilcoxon test. Furthermore, Unweighted and Weighted UniFrac distances (UW-Unifrac and W-Unifrac distances, respectively) were evaluated with PERMANOVA based on 9999 permutations. Comparisons were performed for the six host species in combination, and separating host species by genera, considering the similarity in microbial diversity in amphibians hosts belonging to the same genera [33]. Analyses were done using the packages qiime2R [34], phyloseq [35], ggplot2 [36], and ggpubr [37] in R v 4.0.0 [29].

We examine differences exclusively in salamander skin bacterial communities in function of the type of fragment (previously used for agriculture or livestock), testing for enriched ASVs using LEfSe. Relative abundances of ASVs were the predictor variable and the type of fragment was the response variable, and features with LDA scores >2.0 were considered informative [31]. Furthermore, we performed the ANCOMBC to identify bacteria families of the SBC differentially abundant between the types of fragments. For further analysis we considered an elevational stratification separating lower (< 1900 m asl) and upper (>1900 m asl) cloud forest fragments, to control the elevational variation of our sampling sites. We addressed the differences in observed ASVs, Shannon index, Faith’ s index, and beta diversity dispersion of the SBC by the type of fragments using t-student or Wilcoxon test. Furthermore, we tested differences in UW-Unifrac and W-Unifrac distances by type of fragments using PERMANOVA based on 9999 permutations. Finally, we addressed differences in the alpha and beta diversity metrics mentioned above by type of fragments for the subset of the widely distributed species *Aquiloeurycea cafetalera*.in order to eliminate any taxonomic effects caused by the inclusion of multiple species in the analyses.

We calculated *Bd* prevalence using the Clopper-Pearson confidence intervals [38] to compare it between types of fragments (previously used for agriculture vs. livestock). Due to the low number of *Bd* positive samples we only report prevalence and infection loads and did not perform any additional tests.

## Results

Environmental variation visualized by PCA showed that the types of fragments (previously used for agriculture vs. livestock) differed in their environmental characteristics. The PCA shows that a gradient of environmental temperature and humidity (PC1) explains 40% of the variance between the types of fragments. Furthermore, microhabitat temperature and number of logs (PC2) explain 29% of the variation between fragments (Online Resource 3).

We obtained a total of 188 swab samples (111 from the skin of six salamander species and 77 from the environment) from eight cloud forest fragments in central Veracruz (Online Resource 4). A total of 8,243,449 demultiplexed raw sequences from the 16 S rRNA V4 region were obtained. After quality control, 185 samples were retained (Table 1), yielding 6,393,191 high-quality sequences with an average depth of 34,006 reads per sample. After rarefaction at 10,000 reads, the final dataset comprised 41,997 ASVs across 185 samples.

### Differences Between Environmental and Salamander Skin Bacterial Communities

**Fig. 2:**
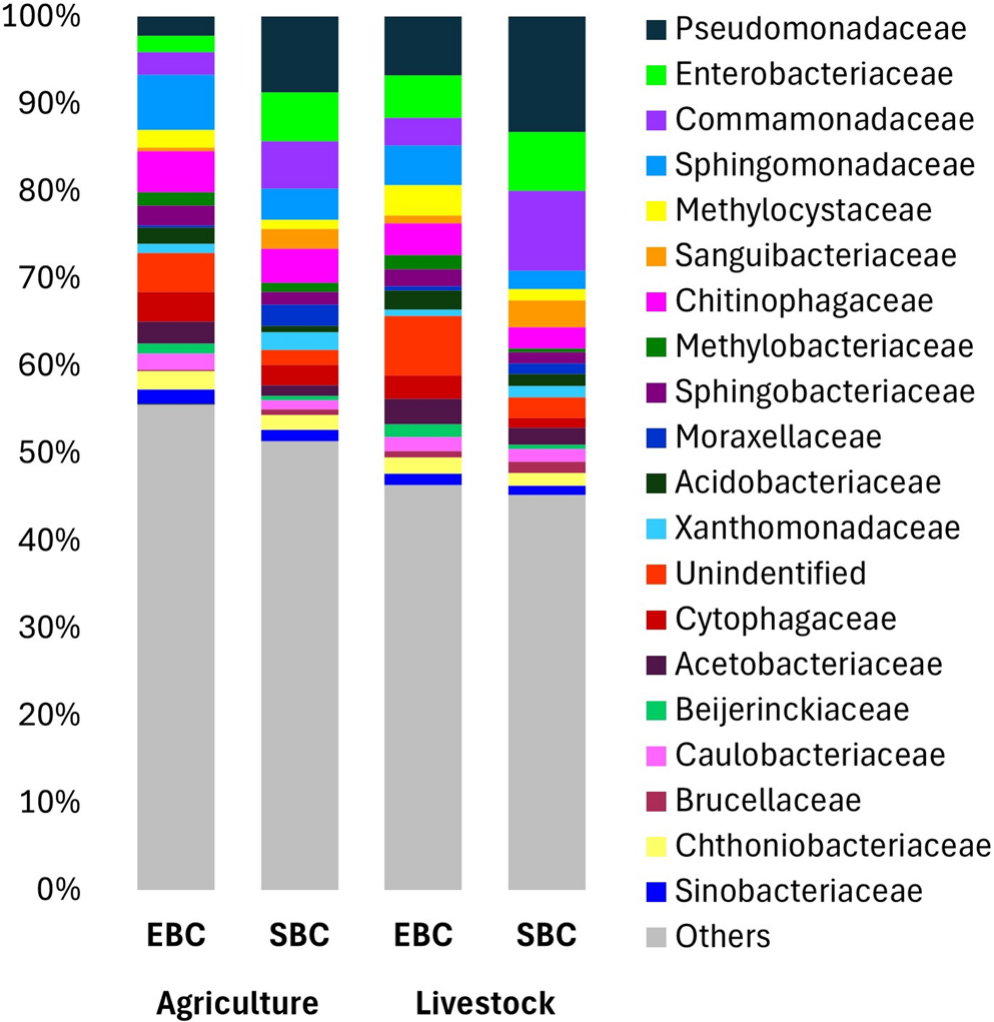
Relative abundances of bacterial taxa at the family level in environmental (EBC) and salamander skin (SBC) communities by type of previous land-use (for agriculture or livestock). Only bacterial families that encompass 50% of the total abundances are shown.

In forest fragments previously used for agriculture, the bacterial families with the highest relative abundance in the environment were Sphingomonadaceae (6.35%), Chitinophagaceae (4.72%), and an unidentified family (4.59%); and in salamanders, the families with the highest relative abundance were Pseudomonadaceae (8.74%), Enterobacteriaceae (5.62%), and Comamonadaceae (5.47%). Meanwhile, in fragments previously used for livestock, families with the highest relative abundance in the environment were the unidentified family (7.04%), Pseudomonadaceae (5.53%), and Sphingomonadaceae (5.37%); and, in salamanders Pseudomonadaceae (11.94%), Comamonadaceae (8.16%), and Enterobacteriaceae (5.95%) (Fig. 2).

In forest fragments previously used for agriculture, we identified eight ASVs that significantly differed between the EBC and SBC: one enriched in the EBC, and seven in the SBC. In the fragments previously used for livestock, 12 ASVs differed between the EBC and SBC: four were enriched in the EBC and eight in the SBC (Online Resource 5). In addition, ANCOMBC identified 42 bacterial families that were differentially abundant between the EBC and SBC in fragments previously used for agriculture, and 47 families were differentially abundant in fragments previously used for livestock (Online Resource 6).

**Fig. 3:**
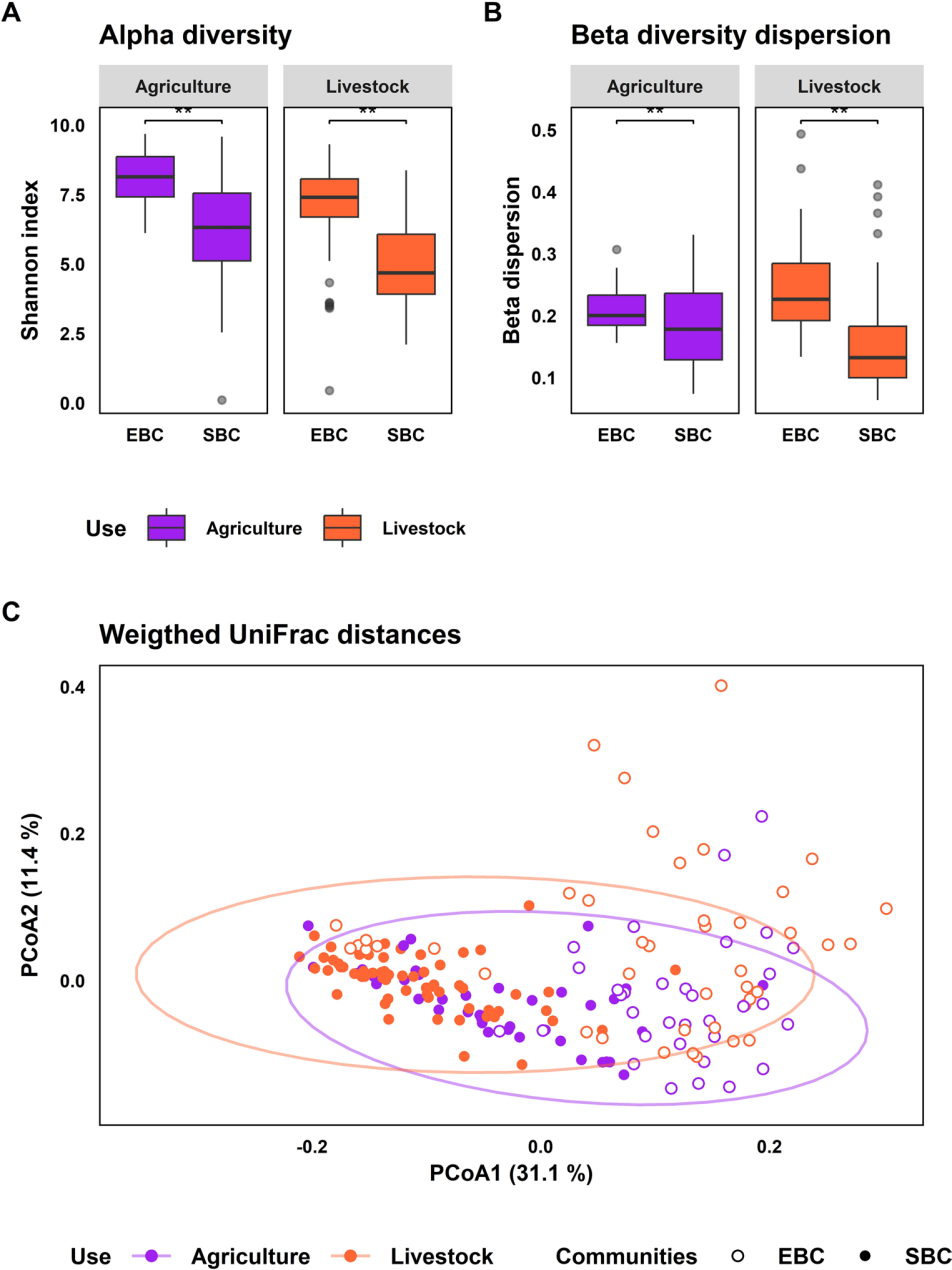
Differences in bacterial diversity from environment and salamander skin separating cloud forest fragments previously used for agriculture and livestock. (**A**) Alpha diversity represented by Shannon index for environmental (EBC) and skin bacterial communities (SBC) in function of the previous land-use. (**B**) Beta diversity dispersion for EBC and SBC in function of the previous land-use. (**C**) Principal coordinate analysis based on Wweighted UniFrac distances of EBC and SBC by previous land-use. The asterisks indicate statistically significant pairwise comparisons (* *p* < 0.01, ** *p* < 0.001)

Regarding alpha diversity, EBC exhibited a significantly higher diversity than the SBC considering all samples (observed ASVs, *X*^2^ = 24.07, *p* value < 0.001; Shannon index, *X*^2^ = 49.17, *p* value < 0.001; Faith PD, *t* = 4.22, *p* value < 0.001). Separately, in fragments previously used for agriculture, EBC showed higher alpha diversity than SBC (observed ASVs, *X*^2^ = 9.31, *p* value = 0.001; Shannon index, *t* = 5.39, *p* value < 0.001; Faith PD, *t* = 2.41, *p* value = 0.018); this pattern was maintained in fragments used for livestock (observed ASVs, *X*^2^ = 14.76, *p* value < 0.001; Shannon index, *X*^2^ = 25.97, *p* value < 0.001; Faith PD, *t* = 3.65, *p* value < 0.001) (Fig. 3A). Comparisons in host genera separately vs. environment showed the same trend seen when all host species were analyzed together (Online Resource 7).

Beta dispersion was higher in EBC than in SBC considering all samples (*t* = 7.04, *p* value < 0.001).This trend was maintained also when samples were separated by previous land-use, EBC had significantly higher beta dispersion than SBC in fragments previously used for agriculture (*t =* 2.64, *p* value = 0.011), and for livestock (*X*^2^ = 17.89, *p* value < 0.001) (Fig. 3B). Comparisons of host genera separately vs. environment showed the same trend as in the analyses of all host species in combination (Online Resource 7). EBC and SBC differed in UW-Unifrac distances (*pseudo*-F = 2.41, *p* value = 0.001) and in W-Unifrac distances (*pseudo*-F = 4.15, *p* value = 0.001) in fragments previously used for agriculture. Bacterial communities also differed in previous land-use for livestock in UW-UniFrac (*pseudo*-F = 4.03, *p* value = 0.001) and in W-UniFrac distances (*pseudo*-F = 19.87, *p* value = 0.001) (Fig. 3C).

### Influence of Historical Land-use on Salamander Skin Bacteria

**Fig. 4:**
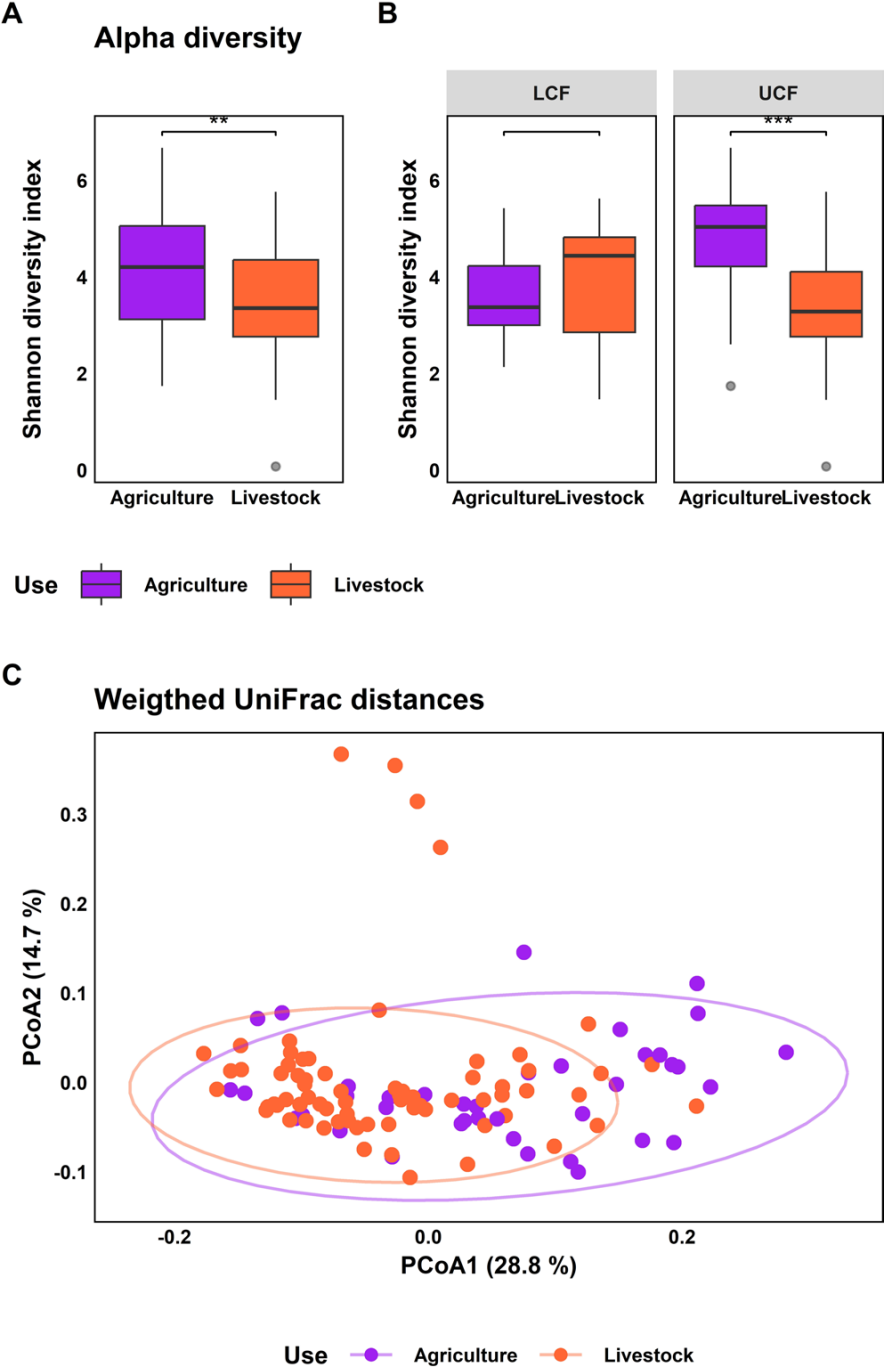
Effect of two types of previous land-use in salamanders’ skin bacterial communities from the secondary cloud forest. (**A**) Alpha diversity represented by Shannon index for previous land-use for agriculture vs. livestock. (**B**) Bacterial alpha diversity across types of previous land-use in upper and lower cloud forest. (**C**) Principal coordinate analysis of the weighted UniFrac distances by type of previous land-use. The asterisks indicate statistically significant pairwise comparisons (* *p* < 0.05, ** *p* < 0.01)

In the SBC, we identified eight ASVs significantly enriched in fragments previously used for agriculture (Online Resource 8), and none enriched in those previously used for livestock. ANCOMBC showed that 18 bacterial families were differentially abundant in fragments previously used for agriculture, and 25 families were differentially abundant in those previously used for livestock (Online Resource 9). Considering all salamander samples, fragments previously used for agriculture had a greater alpha diversity than those used for livestock in three metrics (observed ASVs: *X*² =10.79, *p* value = 0.001; Shannon index: *t* = 2.62, *p* value = 0.011; Faith PD, *t* = 2.92, *p* value = 0.005) (Fig. 4A). Separating fragments by elevation, this pattern was consistent across upper fragments (observed ASVs, *X*^2^ = 14.66, *p* value < 0.001; Shannon index, *t =* 3.79, *p* value = 0.001; Faith PD, *t =* 3.41, *p* value = 0.002) but not in lower cloud forest fragments (observed ASVs, W = 849.5, *p* value < 0.004; Shannon index, *t =* 0.50, *p* value < 0.626; Faith PD, *t =* 1.04, *p* value = 0.318) (Fig. 4B).

Beta dispersion did not differ between the types of fragments considering all salamander samples (*t* = 0.52, p value = 0.605), nor for fragments separated by elevation, in UCF (*t* = 0.52, *p* value = 0.608); and in LFC (*t* = 2.12, *p* value = 0.059). SBC differed by type of fragment in UW-Unifrac (*pseudo*-F = 2.83, *p* value = 0.001), and in W-UniFrac distances (*pseudo*-F = 7.53, *p* value = 0.001) (Fig. 4C). Separately, in the UCF, both beta diversity metrics showed significant differences (UW-Unifrac distances, *pseudo*-F = 2.33, *p* value = 0.001; and W-Unifrac distances, *pseudo*-F = 8.11, *p* value = 0.001), but in the LCF, only UW-Unifrac distances differed significantly (UW-Unifrac distances, *pseudo*-F = 2.39, *p* value = 0.002; W-Unifrac distances, *pseudo*-F = 1.66, *p* value = 0.105).

### Skin Bacterial Diversity on *Aquiloeurycea cafetalera*

**Fig. 5:**
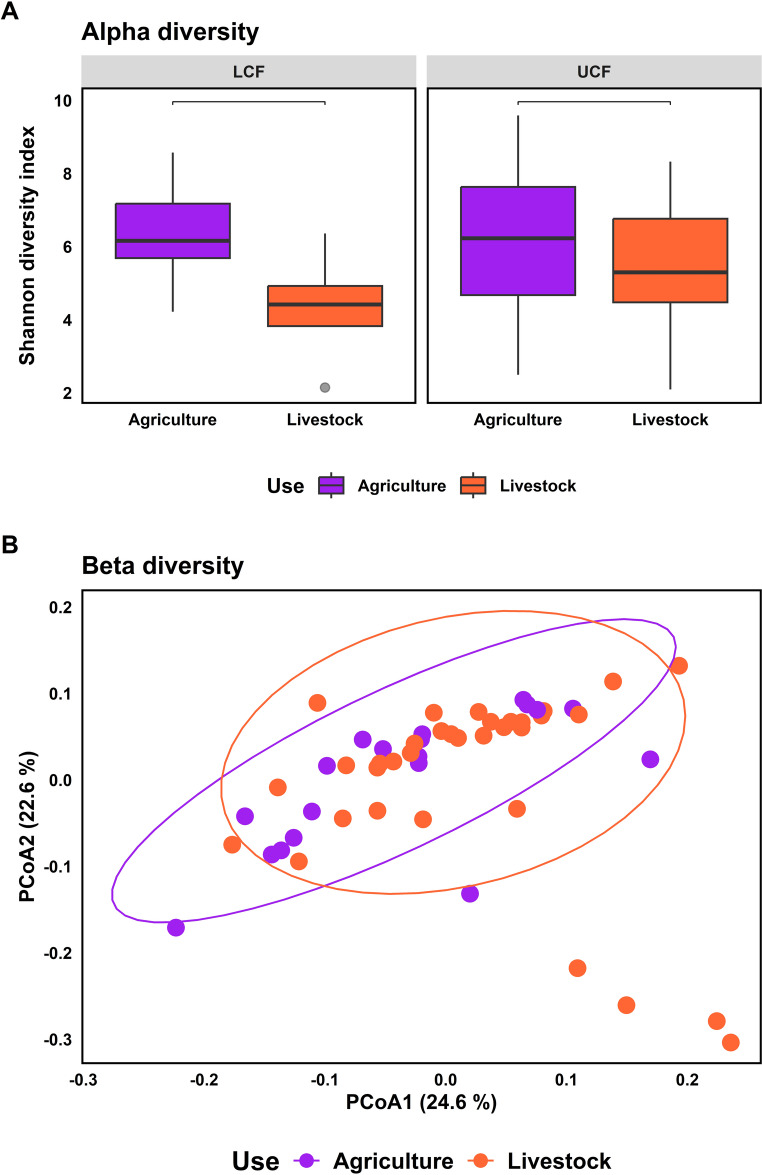
Effect of two types of previous land-use in skin bacterial communities of the species *Aquiloeurycea cafetalera*. (**A**) Bacterial alpha diversity (Shannon index) across types of previous land-use in upper and lower cloud forest. (**B**) Principal coordinate analysis of the Weighted UniFrac distances by type of previous land-use. No significant differences were found in alpha diversity.

Within the widespread species *Aquiloeurycea cafetalera*, alpha diversity did not differ by type of fragment (observed ASVs, *X*^2^ = 2.07, *p* value = 0.151; Shannon index, *t =* 0.37, *p* value < 0.715; Faith PD, *t* = 1.22, *p* value = 0.234). Considering elevation, in the LFC, ASVs richness and phylogenetic diversity did not differ by type of fragment (observed ASVs, *X*^2^ = 1.84, *p* value = 0.174; Faith PD, *t* = 2.10, *p* value = 0.072), but differences were observed in Shannon index (Shannon index, *t* = 5.84, *p* value = 0.002). Meanwhile, in the UCF, bacterial alpha diversity did not differ between types of fragment (observed ASVs, *X*^2^ = 5.34, *p* value = 0.021; Shannon index, *X*^2^ = 2.91, *p* value = 0.088; Faith PD, *X*^2^ = 3.77, *p* value = 0.052) (Fig. 5A).

Considering all *A. cafetalera* samples, beta diversity showed differences between types of fragments (previously used for agriculture and livestock) in UW-Unifrac distances (*pseudo*-F = 2.09, *p* value = 0.004) and W-Unifrac distances (*pseudo*-F = 2.36, *p* value = 0.023) (Fig. 5B). Separately, in the LCF, the SBC differed by type of fragment in UW-Unifrac distances (*pseudo*-F = 1.90, *p* value = 0.009), but not in W-Unifrac distances (*pseudo*-F = 1.59, *p* value = 0.165). In the UCF, SBC differed in UW-Unifrac distances (*pseudo*-F = 1.72, *p* value = 0.007) and in W-Unifrac distances (*pseudo*-F = 2.29, *p* value = 0.023).

### Prevalence and Infection Load of the Pathogen *B. dendrobatidis*

 Out of the 111 swabbed salamanders, only five of them tested positive for *Bd*, yielding an overall prevalence of 4.54%. (95% CI: 1.94–10.29) and a mean infection load of 60,790 ± 135,720 GE. The positive samples came from four localities, and two host species: *Aquiloeurycea cafetalera* (four *Bd* positive samples) and *Parvimolge townsendi* (one *Bd* positive sample). Fragments previously used for agriculture had a prevalence of 2.56% (95% CI: 0.00–13.48%) and previously used for livestock had a prevalence of 5.71% (95% CI: 1.58–13.99%).

## Discussion

In Mexico, the cloud forest is the habitat with the highest amphibian diversity, however, this ecosystem has suffered severe habitat modification affecting amphibian species diversity [39]. In this study, we identified differences in environmental and salamander skin bacterial communities in secondary cloud forests with different previous land-use. Past habitat modification for livestock was associated with differences in composition, lower alpha diversity, and changes in beta diversity in both, environmental and salamanders’ skin bacterial communities. These effects were somewhat consistent across multiple host species and within a single widespread species. Our results indicate that past land-use history can impact free-living and skin bacterial assemblages, depending on the environmental conditions resulting from the history of land-use.

### Habitat Modification Impacts on Environmental and Salamander Skin Bacterial Communities

It is known, from other amphibian systems, that the skin microbiome differs from environmental communities and contains bacteria that carry out specific functions related to amphibian health [2, 5]. Here, we provide evidence that this trend is also present in secondary forests with distinct previous land-use. Despite differences in assembly processes in bacterial communities from soil and amphibian skin [5], both showed lower levels of alpha diversity and higher beta dispersion in areas previously used for livestock. Livestock activities can alter biochemical soil conditions [40], negatively affecting the diversity soil microbial communities, even in the long term [41]. Our results support that ranching has a lasting impact effect even after years of habitat regenetarion [10, 11]. This could explain the enrichment of opportunistic bacteria, such as *Stenotrophomonas maltophilia* [42] observed in the salamanders’ skin from these habitats.

Certain land-use histories in secondary cloud forests with a minor handling appear to have a positive impact on bacterial communities’ diversity. Rainfed agriculture is characterized by use land-use rotation, with long non-handling periods [20], which could improve the nutrients availability for microbe communities [11, 40, 43]. Additionally, the areas previously used for agriculture had higher humidity and lower temperature, conditions that are optimal for the host salamanders [1, 2]. Thus, amphibians in these habitats likely can perform their behavioral and physiological processes that allowed them to host higher diverse bacterial communities [5, 9].

### The Scope of the Impact of Previous Land-use on Symbiotic Bacteria

Secondary cloud forests have been shown to maintain 60–70% of the cloud forest amphibian species, serving as refuges in transformed landscapes [21, 44]. Nonetheless, we observe an influence of the cloud forest land-use history on the amphibians’ bacterial communities, especially at lower elevations. Differences in bacterial communities from lower and upper cloud forests were expected due to bioclimatic variations with elevation [6, 8]. Higher temperatures and lower precipitation in lower elevations can cause physiological stress in amphibians, impacting their interaction with symbiotic bacteria [10, 12], rendering them more vulnerable to opportunistic bacterial taxa [1, 2].

The influence of previous land-use on bacterial communities seems to extend beyond host taxonomic differences on lower scales. Host species and genus are related to differences in composition and structure of the amphibian skin bacterial communities, even in sympatric species [7, 45, 46]. This could explain why some differences in alpha and beta diversity between types of modification were not maintained when we eliminated the effect of multiple host species. On the other hand, the retained differences in bacterial communities suggest that habitat conditions, shaped by land-use history, can have a stronger influence than host phylogeny. For example, we found that bacterial families linked to arboreality in amphibians, such as Alcaligenaceae, were differentially abundant between past land use types, suggesting the relevance of the vegetational structure of the secondary cloud forest resulting from the previous land-use [45]. This aligns with studies highlighting habitat as an important factor shaping microbial communities in plethodontid salamanders [7, 33, 47].

### Pathogen Infection Dynamics in Fragments of Cloud Forest

The low prevalence of *Bd* in this study limits our interpretation regarding the role of history of land-use and skin microbial communities in pathogen dynamics. It has been proposed that low prevalence of *Bd* may be related to intrinsic traits of the plethodontid salamanders, including production of antimicrobial peptides or adaptive immune defenses [1]. On the other hand, the protective role of the microbiome against pathogens remains as a potential explanation for the current low *Bd *prevalence observed in pletodontid salamanders’ communities [33, 47, 48]. Specifically, we found a high abundance of bacterial families with many members known to have potential antifungal properties, such as Comamonadaceae, Enterobacteriaceae, and Pseudomonadaceae [49] exclusively in salamander skin samples. This is interesting, and the potential antimicrobial function of these taxa could be addressed in future studies analyzing the antifungal capacity of plethodontid skin bacteria through culturable techniques or metagenomic inferences.

### Habitat Modification Implication in Future Conservation

Prior research indicates that land-use history leaves persistent ecological legacies on soil and symbiont bacterial communities [9, 41, 43, 50]. Our findings show that the type of land-use practices in the past may have a differential influence in the microbial communities’ diversity. This adds to previous research in the cloud forest by Díaz-García et al. (2020) who emphasized the long-lasting influence of the land-use change on amphibian communities and their functional diversity. Nonetheless, we support the conservation value of secondary cloud forest should not be underestimated [21], and added that this is particularly relevant in lands previously used for agriculture. Previous land use must be considered in the selection of conservation areas, considering that certain land uses may require bioremediation practices and/or longer restoration time.

## Conclusion

This research highlights the variable effects of the land use history on salamander symbiotic bacteria. Agricultural-modified habitats show a greater capacity to host microbial diversity than livestock-modified habitats. The influence of previous or historical habitat modification could be due to the conditions’ alterations along the forest regeneration period and the resulting current environmental conditions. By integrating microbial diversity into conservation frameworks, we can enhance our understanding of ecosystems resilience and develop targeted strategies to safeguard amphibian populations against emerging threats.

## Supplementary Information

Below is the link to the electronic supplementary material.

Supplementary Material 1

## Data Availability

The nucleotide sequence data reported is available at the NCBI SRA under the accession number PRJNA926363.
