# Supplementary material for: Imprints of Land Use History on the Cutaneous Microbiota of Mexican Cloud Forest Salamanders
Source: Microb Ecol. 2025 Dec 5;89:20. doi: 10.1007/s00248-025-02671-5 (PMC12795882; doi:10.1007/s00248-025-02671-5)

Imprints of land use history on the cutaneous microbiota of Mexican cloud forest salamanders

Ángel F. Soto-Pozos[1,4], Eria A. Rebollar[2], Sean M. Rovito[3] & Gabriela Parra-Olea[1,5].

[1]Departamento de Zoología, Instituto de Biología, Universidad Nacional Autónoma de México, AP 70-153, Tercer Circuito Exterior s/n, Ciudad Universitaria, México, Distrito Federal, México. angelfernandosoto@ciencias.unam.mx.

[2] Centro de Ciencias Genómicas, Universidad Nacional Autónoma de México, Cuernavaca, Morelos 62210, México. rebollar@ccg.unam.mx.

[3]Unidad de Genómica Avanzada, Centro de Investigación y de Estudios Avanzados del Instituto Politécnico Nacional, Irapuato, Guanajuato, México. sean.rovito@cinvestav.mx.

[4]Posgrado en Ciencias Biológicas, Unidad de Posgrado, Edificio D, 1° Piso, Circuito de Posgrados, Ciudad Universitaria, Coyoacán, C.P. 04510, CDMX, México

*[5]gparra@ib.unam.mx. Corresponding author**

Online Resource 1. Methodological details

Environmental conditions characterization. At each study site, we set up 1–3 plots of 25 × 25 m in which we recorded: elevation, with a Garmin Etrex 30 GPS; canopy cover with digital photographs of the canopy taken at 20 random points in each plot during high-luminosity diurnal hours; density of trees and logs, by counting the number of trees (diameter >10 cm) and logs per plot; leaf litter depth, with a graduated ruler introduced into the litter on the soil at 20 random points in each plot; environmental temperature and humidity, using a HOBO U23 Pro v2 data logger (Onset, Bourne, Massachusetts, USA) located in the center of one random plot by site for 48 hours; and microhabitat temperature, taken where each salamander was found with a Fluke 62 Max infrared thermometer.

Amplicon library specifications

PCR conditions consisted of initial denaturation at 94 °C for 3 min; 35 cycles of 94 °C for 45 s, 50 °C for 60 s, and 72 °C for 90 s; and a final extension step at 72 °C for 10 minutes. Amplicons were quantified using a QUBIT 4 fluorometer (Invitrogen Thermo Fisher Scientific, Waltham, USA), pooled in equimolar ratios of 240 ng per sample, and purified using the QIAquick PCR Clean-up Kit (Qiagen, Valencia, USA).

Online Resource 2. Shapiro-Wilk normality test results for alpha and beta diversity metrics of the bacterial communities of 76 environmental and 109 salamander skin samples.Numbers in **bold** indicate significant p-values.

	Historical land-use							
	Agriculture				Livestock			
	Environment n= 33		Salamanders n= 39		Environment n= 43		Salamanders n= 70	
Metric	Statistic	p-value	Statistic	p-value	Statistic	p-value	Statistic	p-value
Observed OTUs	0.92041	**0.0188**	0.83667	**0.00005**	0.94687	**0.04586**	0.90309	**0.00013**
Shannon Index	0.96843	0.4381	0.96025	0.18170	0.84968	**0.00005**	0.97199	0.16730
Faith'Index	0.95469	0.1821	0.95225	0.09789	0.98052	0.66790	0.96228	0.05400
Beta dispersion	0.91273	**0.01159**	0.91427	**0.00580**	0.92468	**0.00767**	0.96659	0.89310

Online Resource 3. Principal Component Analysis (PCA) for eight environmental variables on eight sampling sites grouped by the type of cloud forest fragments (previously used for agriculture and livestock).

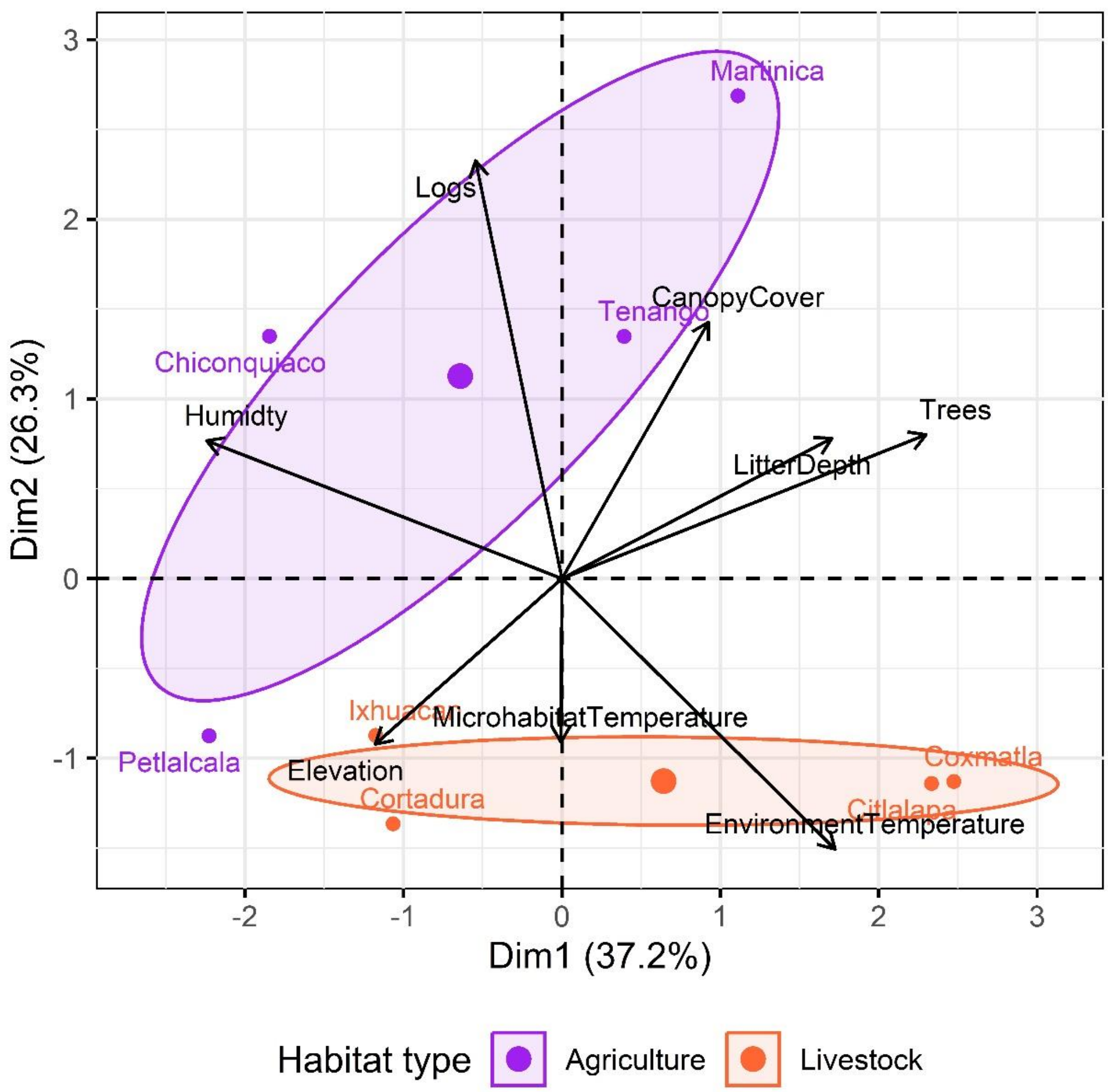

Online Resource 4. Skin sample size by site and by host species, and environmental sample size by site prior to bioinformatic quality filtering. *A.caf= Aquiloeurycea cafetalera, C.nub: Chiropterotriton nubilus, P.tow=Parvimolge townsendi, P.lyn= Pseudoeurycea lynchi, P. gr= P. granitum, P. nig= P. nigromaculata.*

Land-use	Site	*A.caf*	*C.nub*	*P.tow*	*P.lyn*	*P. gr*	*P.nig*	Environment
Agriculture	Chiconquiaco				5			7
	Petlalcala	11				3		12
	Martinica	8		8				10
	Tenango						5	4
	**Total**	**19**		**8**	**5**	**3**	**5**	**33**
Livestock	Cortadura	12	2		15			15
	Coxmatla	6	6	9				12
	Ixhuacán	12			1			11
	Citlalapa	4		4				6
	**Total**	**34**	**8**	**13**	**16**			**44**

Online Resource 5. ASVs differentially abundant between environmental (EBC) and skin bacterial communities (SBC) according to the Linear Discriminant Analysis Effect Size (LefSe) performed separately for bacterial communities from cloud forest fragments previously modified by agriculture and livestock.

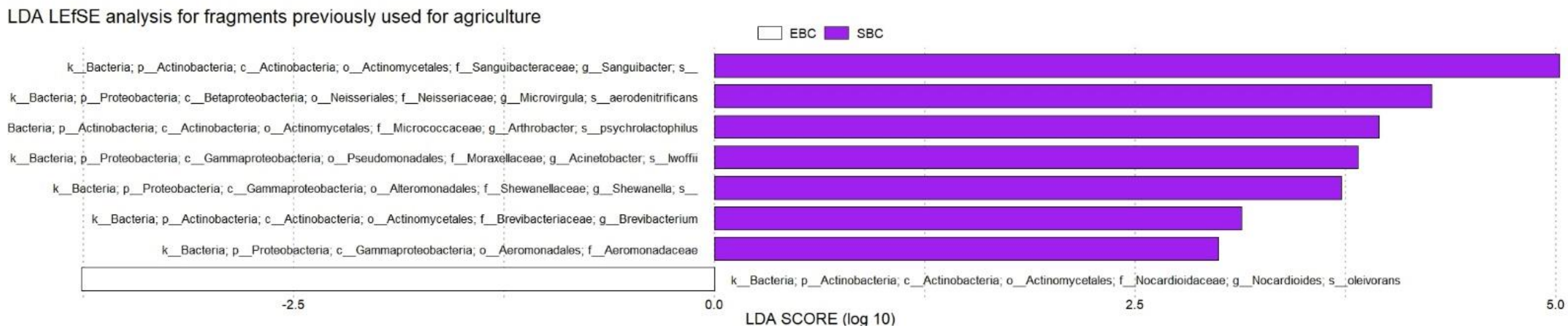

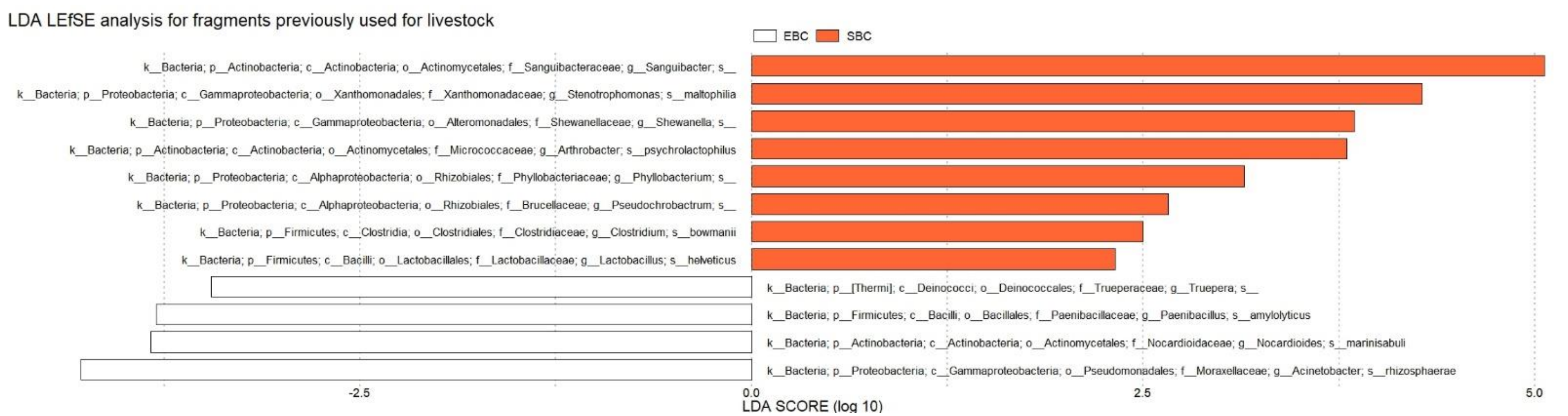

Online Resource 6. Bacterial families with differential abundance in salamander vs environmental samples from cloud forest fragments previously used for agriculture and livestock according to the analysis of compositions of microbiomes with bias correction (ANCOMBC). Left column shows bacterial phylum and family of each taxa. In red are families positively differentiated and in purple negatively.

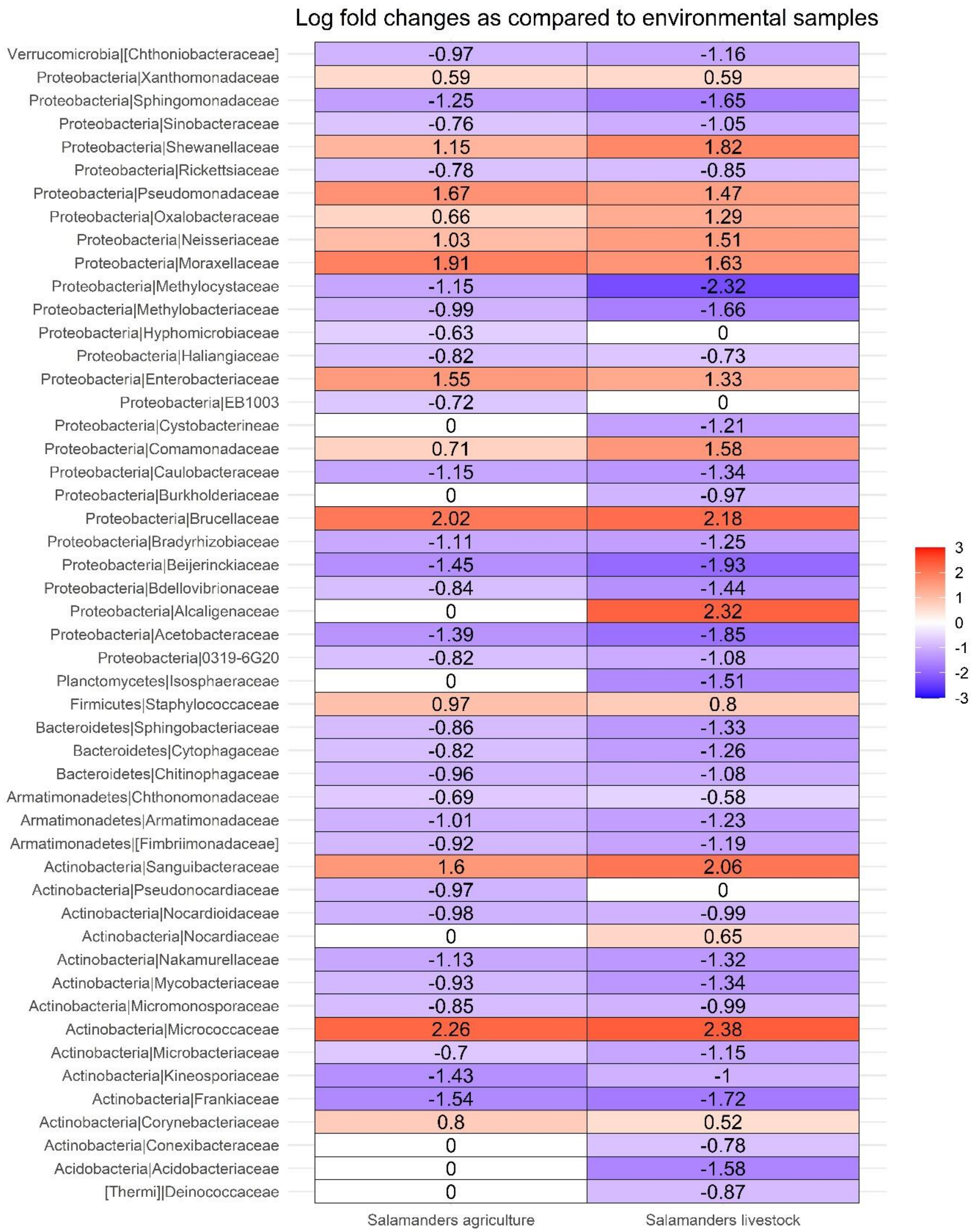

Online Resource 7. Differences in alpha and beta diversity between environmental and skin bacterial communities in two types of fragments (previously used for agriculture and livestock.

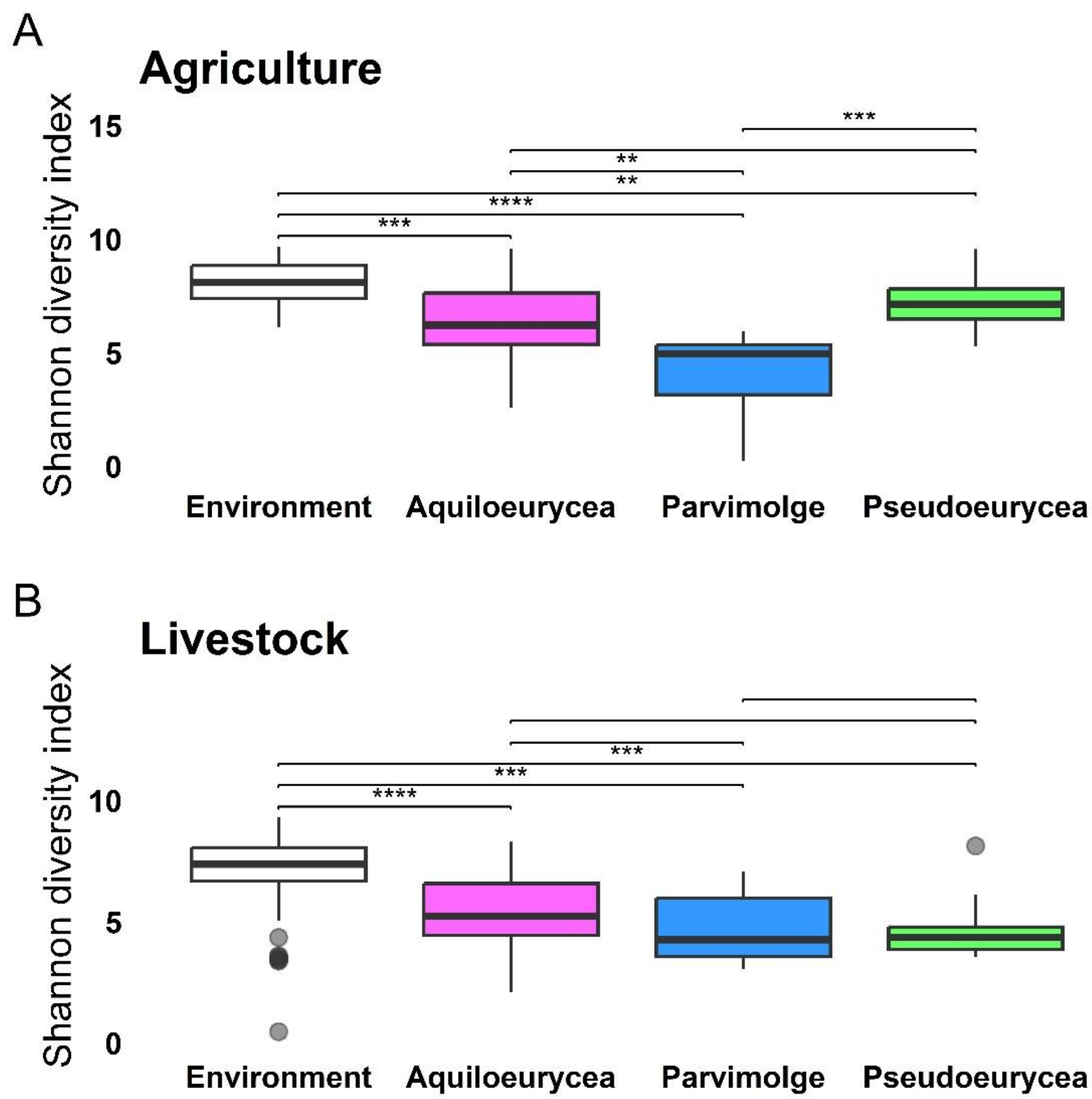

Alpha diversity (Shannon index) differences between bacterial communities from environment vs skin salamanders by host genus (*Aquiloeurycea, Parvimolge* and *Pseudoeurycea*) by type of forest fragments (previously used for agriculture (A) and livestock (B).

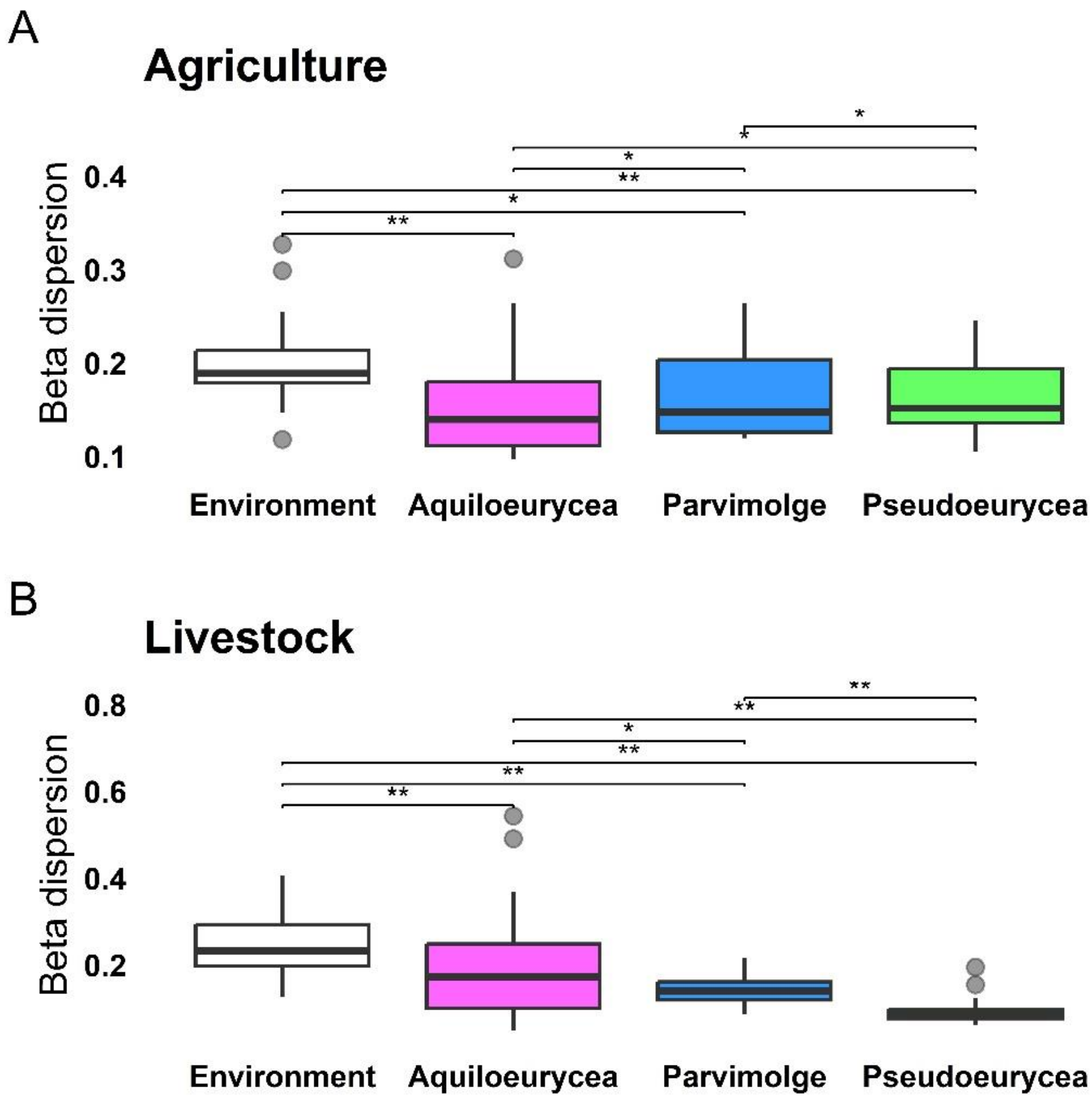

Beta diversity (based in weighted Unifrac distance matrix) differences between bacterial communities from environment vs skin salamanders by host genus (*Aquiloeurycea, Parvimolge* and *Pseudoeurycea*) by type of forest fragments (previously used for agriculture (A) and livestock (B).

Online Resource 8. ASVs significantly enriched in cloud forest fragment previously used for agricultural activities according to the Linear Discriminant Analysis Effect Size (LefSe) performed for the skin bacterial communities of pletodontid salamanders.

**ASVs**	**scores**
p__Proteobacteria; c__Gammaproteobacteria; o__Pseudomonadales; f__Moraxellaceae; g__Acinetobacter; s__schindleri	-4.123776
p__Proteobacteria; c__Gammaproteobacteria; o__Enterobacteriales; f__Enterobacteriaceae; g__Serratia; s__marcescens	-4.114578
p__Proteobacteria; c__Alphaproteobacteria; o__Caulobacterales; f__Caulobacteraceae; g__Brevundimonas; s__staleyi	-3.867359
p__Proteobacteria; c__Gammaproteobacteria; o__Xanthomonadales; f__Xanthomonadaceae; g__Arenimonas; s__oryziterrae	-3.766932
p__Actinobacteria; c__Actinobacteria; o__Actinomycetales; f__Cellulomonadaceae; g__Cellulomonas; s__xylanilytica	-3.749934
p__Actinobacteria; c__Actinobacteria; o__Actinomycetales; f__Kineosporiaceae; g__Quadrisphaera; s__granulorum	-3.696743
p__Actinobacteria; c__Actinobacteria; o__Actinomycetales; f__Nocardioidaceae; g__Marmoricola; s__aequoreus	-3.568691
p__Actinobacteria; c__Actinobacteria; o__Actinomycetales; f__Microbacteriaceae; g__Rathayibacter; s__caricis	-3.312283

Online Resource 9. Bacterial families with differential abundance in fragments previously used for agriculture vs livestock in the salamanders' skin bacterial communities according to the analysis of compositions of microbiomes with bias correction (ANCOMBC).

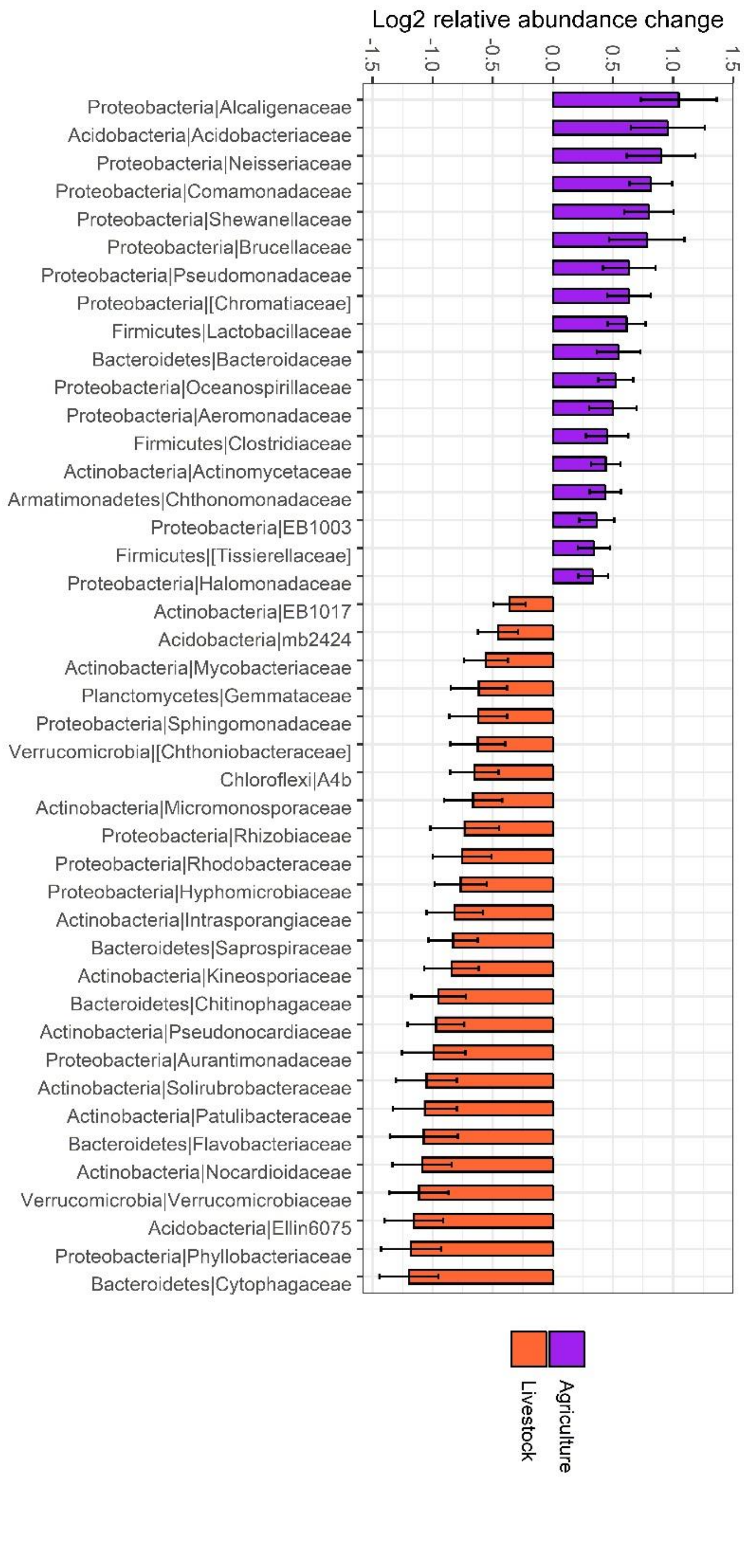

Supplement: Supplementary file 1 [file 248_2025_2671_MOESM1_ESM.pdf]
